# Recent Advances in Crop Genetics and Breeding

**DOI:** 10.3390/life15121891

**Published:** 2025-12-11

**Authors:** Tianxiao Chen, Xiangjin Wei, Baohua Feng, Long Chen

**Affiliations:** State Key Laboratory of Rice Biology and Breeding, China National Rice Research Institute, Hangzhou 310006, China

Crop improvement remains central in addressing global challenges related to food security, climate change, and sustainable agriculture. Advances in genomics, high-throughput phenotyping, bioinformatics, and gene-editing technologies are reshaping modern crop breeding strategies. This Special Issue, *“Recent Advances in Crop Genetics and Breeding”*, in *Life* brings together six research articles that collectively highlight emerging insights into genetic architecture, regulatory networks, germplasm diversity, and innovative breeding methodologies. This editorial summarizes the major findings of these contributions, discussing their significance within the broader context of crop improvement.

## 1. The Genetic Basis of Rice Root System Architecture Under Salt Stress

Xue et al. conducted a genome-wide association study (GWAS) using 165 rice accessions from the 3K Rice Genome Project to dissect root system architecture (RSA) responses under salt stress (contribution 1). Using detailed imaging and the 3VmrMLM model, they identified 78 QTNs correlated with eight RSA traits, including root volume and surface area. Several loci overlapped with previously reported salt-tolerance genes, with six accessions exhibiting superior salt-induced root performance. This study demonstrates how precise root phenotyping can uncover key loci for breeding salt-tolerant rice, emphasizing RSA’s importance in stress adaptation.

## 2. A Transcriptomic Meta-Analysis of Wild Relatives and Domesticated Crops

Yumiya and Bono performed a comparative meta-analysis of transcriptome datasets from wild relatives and domesticated varieties of rice, tomato, and soybean (contribution 2). They observed that wild species consistently show higher stress-responsive gene expression, while domesticated cultivars exhibit increased expressions of hormone- and detoxification-related genes. The authors propose integrating wild germplasm and de novo domestication strategies through modern gene-editing approaches. Their study underscores the value of wild relatives in enhancing stress tolerance and genetic resilience, offering an evolutionary perspective on domestication.

## 3. OsMYBR1 as a Regulator of Rice Endosperm Starch Biosynthesis

Huang et al. identified the 1R-MYB transcription factor *OsMYBR1* as a key regulator of starch biosynthesis during rice endosperm development (contribution 3). Loss-of-function mutants displayed altered starch granule morphology, reduced grain thickness, and changes in physicochemical properties, whereas overexpression improved eating and cooking quality. *OsMYBR1* activates the transcription of several key starch biosynthetic genes, including *OsGBSSI*, *OsBEIIb*, and *OsISA1*. This work enhances our understanding of transcriptional grain quality regulation, providing promising targets for breeding high-quality rice.

## 4. A Multi-Environment Evaluation of Lentil Germplasm

Sardar et al. assessed 649 lentil accessions over multiple seasons and geographic regions, analyzing yield and related traits through multivariate and stability analyses (contribution 4). They identified high-yielding and environmentally stable accessions and highlighted trait combinations associated with productivity, such as harvest index and pod attributes. Their study provides a robust phenotypic dataset for lentil improvement and illustrates the importance of multi-environment testing in breeding programs.

## 5. Improving Soybean Transformation Efficiency Through Genetic Hybridization

Khan et al. developed soybean hybrid lines with improved Agrobacterium-mediated transformation efficiency by combining susceptible alleles from Williams82 with high-flavonoid backgrounds (contribution 5). Optimized co-cultivation conditions and genotype selection led to lines with significantly higher regeneration and transgene expression efficiencies. Improving transformation efficiency is a critical bottleneck in soybean biotechnology. This study offers practical strategies that are applicable to functional genomics and gene-editing pipelines.

## 6. The Functional Characterization of GmGASA1-like in Soybean Growth and Stress Adaptation

Khalifa et al. demonstrated that *GmGASA1-like* overexpression influences plant architecture and enhances stress-responsive gene expression under heat, cold, and drought conditions (contribution 6). Some agronomic traits were positively affected, indicating the potential role for GASA family genes in breeding stress-resilient soybeans. The findings highlight multifunctional regulators integrating developmental and environmental cues in crop plants.

Precise measurement technologies, such as the root imaging used in the rice RSA study, are revealing genetic determinants that traditional phenotyping often overlooks. High-throughput phenotyping will increasingly drive more accurate trait–genotype associations. Research on *OsMYBR1* and *GmGASA1-like* highlights transcriptional regulators as central nodes in the pathways governing grain quality, yield components, and stress responses. Dissecting these networks will support the development of predictive models for trait improvement. A transcriptomic comparison between wild and domesticated crops reinforces the relevance of wild relatives in providing adaptive alleles. This aligns with global initiatives aimed at de novo domestication and introgression breeding. Efficient genetic transformation, as shown in the soybean study, is fundamental for gene validation and genome editing. Continued innovation in genotype selection, tissue culture optimization, and delivery systems is crucial for expanding the scope of molecular breeding. The lentil multi-environment study illustrates how detailed phenotypic evaluation forms the foundation of genomic studies such as GWAS or genomic selection. Combining phenomics with genomic prediction will accelerate breeding cycles in diverse crops.

Building on the insights gained from this Special Issue, several key areas warrant further emphasis:

### The Expansion of High-Throughput, Non-Destructive Phenotyping

Integrating imaging technologies, machine learning, and field-based sensors will enhance trait discovery and accelerate breeding decisions.

### The Comprehensive Reconstruction of Regulatory and Metabolic Networks

Multi-omics integration—including transcriptomics, proteomics, epigenomics, and chromatin profiling—will improve our understanding of trait regulation.

### The Strategic Utilization of Wild Relatives and Landraces

Sequencing efforts and targeted gene-editing approaches can unlock valuable alleles for stress resilience and yield stability.

### The Development of Genotype-Independent Transformation and Editing Systems

Broadening transformation applicability across cultivars and species will democratize biotechnology-driven breeding.

### Cross-Disciplinary Collaboration and Data Sharing

Collaboration among geneticists, breeders, bioinformaticians, and climate scientists will strengthen predictive breeding frameworks.

##  Concluding Remarks

The six contributions in this Special Issue collectively advance our knowledge of genetic diversity, regulatory mechanisms, stress adaptation, and biotechnology in crops. They highlight how integrative approaches—combining genomics, phenomics, transcriptomics, and targeted editing—are driving innovation in modern crop breeding. We hope that these studies inspire further research aimed at developing high-yielding, climate-resilient, and nutritionally enhanced crop varieties.

